# Enlisting the mRNA Vaccine Platform to Combat Parasitic Infections

**DOI:** 10.3390/vaccines7040122

**Published:** 2019-09-20

**Authors:** Leroy Versteeg, Mashal M. Almutairi, Peter J. Hotez, Jeroen Pollet

**Affiliations:** 1 Departments of Pediatrics, National School of Tropical Medicine, Baylor College of Medicine, One Baylor Plaza, BCM113, Houston, TX 77030, USA; 2 Texas Children’s Hospital Center for Vaccine Development, Baylor College of Medicine, 1102 Bates Street, Houston, TX 77030, USA; hotez@bcm.edu; 3 Cell Biology and Immunology Group, Wageningen University & Research, De Elst 1, 6708 WD Wageningen, The Netherlands; 4 Prince Naif Health Research Center, King Saud University, Riyadh 11451, Saudi Arabia; mmalmutairi@KSU.EDU.SA; 5 Department of Pharmacology and Toxicology, College of Pharmacy, King Saud University, Riyadh 11451, Saudi Arabia; 6 Vaccines and Biologics Research Unit, College of Pharmacy, King Saud University, Riyadh 11451, Saudi Arabia; 7 Departments of Pediatrics and Molecular Virology & Microbiology, National School of Tropical Medicine, Baylor College of Medicine, One Baylor Plaza, BCM113, Houston, TX 77030, USA; 8 Hagler Institute for Advanced Study at Texas A&M University, College Station, TX 77843, USA; 9 Department of Biology, Baylor University, Waco, TX 76798, USA

**Keywords:** messenger RNA, multivalent vaccines, CD8+ T cells, neglected tropical diseases

## Abstract

Despite medical progress, more than a billion people still suffer daily from parasitic infections. Vaccination is recognized as one of the most sustainable options to control parasitic diseases. However, the development of protective and therapeutic vaccines against tropical parasites has proven to be exceptionally challenging for both scientific and economic reasons. For certain parasitic diseases, traditional vaccine platforms are not well-suited, due to the complexity of the parasite life cycles and the parasite’s ability to evade the human immune system. An effective anti-parasite vaccine platform needs to have the ability to develop and test novel candidate antigens fast and at high-throughput; it further needs to allow for multivalent combinations and must evoke a strong and well-defined immune response. Anti-parasitic vaccines need to be safe and economically attractive, especially in the world’s low- and middle-income countries. This review evaluates the potential of in vitro transcribed mRNA vaccines as a new class of preventive and therapeutic vaccine technologies for parasitic infections.

## 1. Introduction

### 1.1. A Pressing Need for Vaccines Against Parasitic Diseases

Over the last few decades, vaccines have eliminated and reduced numerous infectious diseases. In 1980, smallpox became the first infectious disease to be eradicated thanks to an effective vaccine [1]. Polio cases have been declining since the WHO Global Polio Eradication initiative started in 1988, and cases are at a record low number. As a result, polio is now almost eradicated [2]. Other diseases, like measles, diphtheria, tetanus, rubella, and mumps, have seen a significant reduction in incidence and mortality since the introduction of vaccines [3].

In contrast to the public health gains from vaccinating children against virus and bacterial agents of disease, so far, human parasitic infectious diseases remain a major burden and have largely resisted successful vaccine development efforts. Soil-transmitted helminths and schistosomes are thought to affect as much as a quarter of the world’s population [4]. Protozoa that infect humans can cause severe disease (malaria and kinetoplastid infections, including Chagas disease, leishmaniasis, sleeping sickness) [5]. Shown in Table 1 are some of the most important human parasitic diseases and their disease burden in terms of global prevalence, disability-adjusted life years (DALYs), and deaths, as recently estimated by the Global Burden of Disease Study (2017), an initiative of the Institute of Health Metrics and Evaluation (IHME) and the Gates Foundation. Altogether, it is estimated that nearly two billion people worldwide are infected with at least one (neglected) tropical parasitic disease, while many of these same individuals are “poly-parasitized” with multiple parasitic diseases [6]. Their health impact is substantial. Malaria is a leading cause of death in resource-poor nations, especially in sub-Saharan Africa, while other parasitic diseases exert their adverse health effects by causing profound disability as measured in DALYs [7]. Still another effect is that the disabling features of these parasitic infections often translate to reduced economic productivity so that they actually thwart economic achievements and gains [8]. Finally, it can be noted that while neglected parasitic infections dominate in resource-poor countries, there is increasing evidence for their high prevalence rates among poor and indigenous populations living in wealthier countries, including the United States, Europe, and Australia, a phenomenon sometimes referred to as “blue marble health” [9].

While for all parasitic infections listed in Table 1 specific treatment options like antihelminthic, antitrypanosomal, and other antiparasitic drugs are available, there are often issues preventing their successful application in endemic regions, such as severe side-effects, low-efficacy, drug-resistance, and reinfection. Nontheless, the mainstay of parasitic disease control globally has relied on large-scale mass treatment programs and allied measures. A package of essential anti-parasitic medicines now reaches more than one billion people annually for the treatment of soil-transmitted helminth infections, schistosomiasis, lymphatic filariasis, and onchocerciasis. In the case of lymphatic filariasis and onchocerciasis, this approach may lead to the elimination of these diseases as a public health program in the coming decade, with additional or collateral benefits for additional neglected infections, including scabies [11]. However, for hookworm infection and schistosomiasis, there is an expectation that additional control tools, such as vaccines, will be required in order to effect elimination efforts [12,13]. Similarly, for malaria, tremendous gains have been achieved through the administration of antimalarial drugs and insecticide-treated bed nets, but a vaccine will still be required to go the last mile for this ancient scourge. We urgently need a new generation of vaccines for high-prevalence parasitic infections, such as malaria, leishmaniasis, Chagas disease, hookworm infection, and schistosomiasis [14]. However, there are only a handful of licensed vaccines against parasites available and, with the exception of the malaria vaccine, Mosquirix (RTS, S), which was approved by the European Medicine Agency (EMA) in 2015 and is just now being introduced in three African nations [15], all are for veterinary applications [16]. If parasitic infections cause such a large worldwide burden and vaccines can offer a solution, then why are there no vaccines available?

### 1.2. Why Does Commercial Vaccine Development Steer Clear of Parasitic Infectious Diseases?

The relatively low interest in the commercial development of vaccines for parasitic diseases can be explained by the following factors:

#### 1.2.1. Lack of Knowledge of the Biological Complexity of Parasites

Eukaryotic parasites have large and complex genomes that can challenge the successes of the reverse vaccinology programs now benefiting the development of vaccines to prevent “small genome” bacterial and viral infections. Moreover, many parasites have complex life cycles. Several have developed sophisticated strategies to evade and modulate the host immune system, and sometimes they even exist as different stages within one host [17]. There is often poor knowledge on how the parasite evades the immune system, what the function of specific parasite proteins is, and what antigens should go into an effective vaccine, yielding a protective immune response.

#### 1.2.2. Parasitic Infections Mainly Impact Poor People in Regions of Low Economic Power

These infections often occur in tropical countries with weak economies. Mostly, the poorest of the poor are affected because they typically live in inadequate conditions with poor hygienic conditions and an increased risk of exposure to insect and other disease vectors [18]. In addition, access to healthcare in these regions is often limited, making the development of anti-parasitic vaccines even more important. When parasitic infections do occur in wealthier countries, they disproportionately affect impoverished or indigenous populations who are often not prioritized by government leaders. The term “antipoverty vaccines” has been invoked to describe neglected parasitic disease vaccines because of their simultaneous impact on both public health and economic development [18]. However, from an investment perspective, the fact that these technologies would primarily benefit the poor has had a chilling effect on the traditional investment community targeting new pharmaceuticals.

#### 1.2.3. Most Parasites Cause Chronic Disease and Disabilities but Do Not Kill the Host

Co-evolution between parasites and their hosts have made them capable of establishing chronic infections that can last for decades [19]. Only malaria is a significant killer. As a result, parasitic infections are either not recognized or underestimated for the severe burden they cause.

#### 1.2.4. Limitations of the Traditional Vaccine Platforms

Because of the complexity of parasitic infections, conventional vaccine platforms, such as live attenuated, killed whole parasite or subunit vaccines, including recombinant protein strategies, may not always be effective. Vaccine development for parasitic infections is often hindered by limitations of production and/or inadequate immune responses [20]. There is also the expense associated with traditional vaccine platforms, which might not be linked to a traditional return on investment [14].

From the bench to the clinic, a multitude of attempts have been made to develop efficacious human vaccines for parasitic diseases. Although many anti-parasitic vaccine candidates showed promising results in preclinical models, they either lacked protective capacity in the field or experienced other issues. For example, while a prophylactic live-attenuated vaccine against leishmaniasis showed great protection, it was discontinued due to safety problems because one vaccinated individual presented primary lesions after vaccination [21,22]. A leading recombinant malaria vaccine candidate, MSP-1^42^, tested in children in Kenya, induced high antibody titers but failed to protect against infection [23,24], while Mosquirix is only partially protective [15]. It was found that malaria parasites altered dendritic cell functionality and weakened their ability to support memory B cell survival.

### 1.3. Enlisting mRNA Vaccine Technology to Control Parasitic Diseases

To find a vaccine-based solution for parasitic infections, novel vaccine platform technologies need to be considered. In this review, we discuss the application of in vitro transcribed (IVT) mRNA vaccines for the development of novel vaccines against parasitic infections. We will give a comprehensive introduction of the mRNA vaccine platform with relevance to making mRNA vaccines against parasitic infections. We will discuss the potent immune response of exogenous RNA and highlight the platform’s advantages and limitations for each of the critical vaccine development topics (production, formulation, immunology, stability, and safety). Moreover, we will go over some recently published studies on mRNA vaccines for parasitic diseases and briefly introduce our development plan for an mRNA vaccine to protect against Chagas disease. An accompanying manuscript in this special issue (Poveda et al.) [25] reviews the necessary product characterizations for the initial evaluation of mRNA vaccine antigen candidates, and for more detailed information on the mRNA technology beyond the application for parasitic diseases, we refer the reader to a few excellent recent reviews on the topic [26,27,28,29,30,31].

## 2. Messenger RNA Vaccine Technology

### 2.1. Design and Development of In Vitro Transcribed mRNA

Messenger RNA vaccines apply IVT mRNA as a blueprint to produce vaccine antigens in vivo, in a patient. The translated pathogen-specific antigens will induce a specific immune response, depending on the type of the cell that was transfected, and the immunogenicity of both the mRNA product and the encoded antigen.

**Figure 1:**
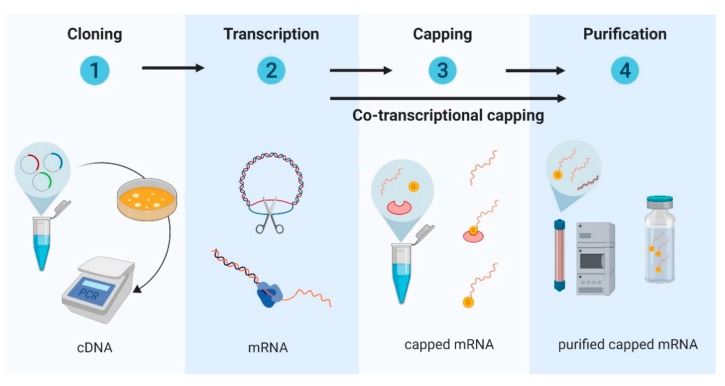
Messenger RNA production steps: (1) DNA construct is cloned in *Escherichia coli* (*E. coli*) bacteria, then purified and amplified; (2) linearized DNA constructs are transcribed; (3) transcripts are either capped during transcription or capped post-transcription; and (4) purified by chromatography.

In vitro mRNA synthesis usually starts with the cloning of the target antigen into a DNA plasmid and its subsequent linearization, although PCR products and synthetic oligonucleotides can also serve as templates for a cell-free in vitro transcription reaction with recombinant RNA polymerase and nucleoside triphosphates [30]. After transcription, the DNA template is removed using RNase-free DNases, and in order to increase mRNA stability and translation efficiency, the transcriptional product is enzymatically capped. Finally, the mRNA product is purified to remove any remaining DNA template, double-stranded RNA, and other contaminations by HPLC [32,33] (Figure 1). The quality of the mRNA transcript is a critical factor, requiring testing for stability, integrity, identity, purity, and homogeneity, as is testing for the desired innate immune response prior to animal testing [25].

**Figure 2:**
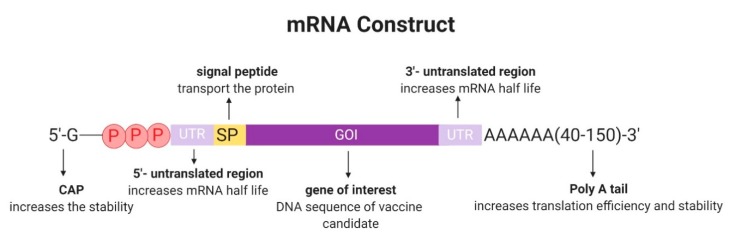
A typical mRNA construct with supporting untranslated regions, poly(A) tail, and an optional signal peptide sequence attached to the coding sequence.

Typically, IVT mRNA is comprised of a protein-encoding open reading frame (ORF) flanked by two untranslated regions (UTRs), to support translation (Figure 2). A signal peptide (SP) may be added to the ORF to facilitate the secretion of the encoded vaccine antigen candidate. The transcript should further contain a 3′ poly(A) tail to improve intracellular stability and translational efficiency. It has been shown that while an increase in poly(A) tail length generally enhances the efficiency of polysome formation, leading to improved protein expression [31], there appears to be an optimal length of 120 and 150 nucleotides [34]. At the 5′ end, the RNA is capped with 7-methyl-guanosine triphosphate (m7G) to protect against RNases [35,36]. Because of the presence of two free 3′-OH on both guanine moieties of the cap structure, approximately one-third of the mRNAs have a cap incorporated in the reverse orientation. These reverse cap structures bind poorly to eIF4E, the cap-binding protein, and therefore decrease translational efficiency [35]. In order to solve the problem, anti-reverse cap analogs (ARCAs) have been developed. ARCAs have only one 3′-OH group, which inhibits the incorporation in the reverse orientation seen with cap analogs. Further improvement of the capping efficiency was made by CleanCap^®^, a co-transcriptional capping method. CleanCap^®^ yields a naturally occurring Cap1 structure, which results in an increased mRNA transcription efficiency compared with ARCA cap analogs [37].

**Figure 3:**
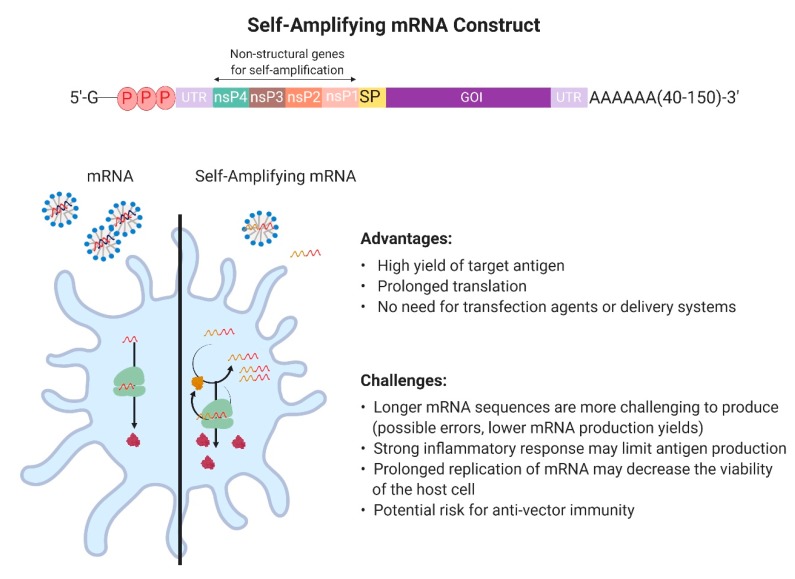
Top: self-amplifying mRNA construct: the sequences of four non-structural proteins (nsPs) from an alpha virus are inserted between the first untranslated region (UTR) and the gene of interest (GOI); bottom left: Schematic of a cell transfected with either a regular mRNA vaccine or a self-amplifying mRNA, illustrating the higher yield of translated protein by the self-amplifying construct; bottom right) Advantages and disadvantages of self-amplifying mRNA.

Multiple groups have developed self-replicating or replicon mRNA vaccine products in order to make mRNA transfection more efficient [38,39]. The construction of a self-amplifying mRNA includes non-structural parts of the genome of positive-strand viruses, e.g., sindbis virus, Semliki Forest virus, or Venezuelan equine encephalitis virus [40], which encode for their own RNA replication system [41,42] (Figure 3). The viral structural protein sequences are replaced with the sequence of the antigen of interest. This mRNA platform has the capability of self-replication through the synthesis of an RNA-dependent RNA polymerase complex, leading to multiple copies of the antigen-encoding mRNA and higher levels of the heterologous target antigen. In a specific study on mRNA vaccines against influenza, self-amplifying mRNA vaccines produced high antibody titers in vivo after injecting only 50 ng mRNA, an amount that is 40 times lower than that used for conventional transfections [43]. While delivery and transfection systems can be used to improve the stability and the cellular uptake of self-amplifying RNA, an advantage of self-amplifying mRNA vaccines is that they do not strictly require an additional delivery system. Due to the design of the alphavirus vectors and its larger size (9–11 kilobases), naked self-amplifying RNA is picked up by mostly antigen-representing cells [38]. It should be noted that the production and purification of longer mRNAs is more challenging and that amplification of mRNA in the host cell can result in a strong inflammatory response, limiting antigen production [44]. In addition, a memory immune response against the replication proteins may limit repeated use, similar to other vaccine platforms applying viral vectors [26].

### 2.2. Messenger RNA Delivery

Naked mRNA can be taken up by many cell types, and has been used successfully for in vivo transfection after intranodal and intradermal administration [26,28,45]. However, with the exception of self-amplifying mRNA (see the previous section), transfection with naked exogenous mRNA is generally not very effective when administered through classic vaccination routes. From the sequences that are taken up by the cells, only a fraction will get into the cytoplasm and most of the internalized mRNA will get entrapped and degraded in lysosomes. In vivo cell transfection can be significantly improved by specific mRNA delivery vehicles and transfection systems [26]. These transfection agents help the exogenous mRNA escape from the endosome into the cytoplasm before being degraded in a lysosome. In addition, the physicochemical properties of the mRNA-transfection complex can influence cellular delivery and organ distribution. Other recent reviews on the mRNA vaccine platform have given a comprehensive and complete overview of current mRNA delivery and transfection systems [43,46]. The most used systems are in vivo electroporation, protamine, cationic nanoemulsion, modified dendrimer nanoparticles, cationic liposomes, cationic polysaccharide particles, cationic polymers, and different versions of cationic lipid nanoparticles (LNPs) [26,47], many of which are now commercially available.

The site of injection is equally important. Multiple injection routes have been explored in the literature [26,30], and in clinical trials, mRNA vaccines have been delivered intradermally, intravenously, and intramuscularly (Clinical trial ID NCT02241135, NCT03014089, NTC03076385, NCT03325075, NCT03345043, NCT03382405, NCT03392389, NCT03713086).

### 2.3. Immune Profile of mRNA Vaccines

Recent research has dramatically improved our understanding of immune activation and immune response induced by mRNA and mRNA vaccines [32,48,49,50,51,52]. The innate immune response has been studied extensively because exogenous mRNA triggers the same host cell receptors as triggered by an RNA virus infection [48,53]. It has been demonstrated that mRNA vaccines can elicit strong CD8+ T cells responses, which are essential in targeting intracellular pathogens [51]. Type I interferons, released through innate immune activation, have been shown to be important activation markers in this context. In addition, a potent CD4+ T cell response has proven to be essential in supporting CD8+ T cell activation and the activation of B cells to differentiate into antibody-secreting plasma cells and memory B cells.

#### 2.3.1. Induction of the Innate Immune Response by mRNA Vaccines

Two major groups of RNA sensors have been identified that are involved in activation of the innate immune system upon mRNA recognition, Toll-like receptors (TLRs) and retinoic acid-inducible gene-I (RIG-I) receptors. TLRs recognize viral agents before the infection is even established, while the RIG family is triggered when viral agents are present in the cytoplasm and viral infection has been established [54]. TLR3, TLR7, and TLR8 are all located in the endosomal compartment of antigen-presenting cells (APCs), including dendritic cells, macrophages, and monocytes. TLR3 detects double-stranded RNA and is the only TLR that acts through the NF-κB pathway, while all the other TLRs act through the MyD88-dependent signaling cascade [55]. TLR7 and TLR8 both detect single-stranded RNA. All pathways lead to the production of type I interferons (IFNα/β) [48]. RIG-I-like receptor family (RLR), located in the cytoplasm, detects single- and double-stranded RNA. LGP2 (Laboratory of Genetics and Physiology 2) is an RLR that has shown to be important in antiviral signaling [56]. MDA5 (melanoma differentiation-associated protein 5) detects double-stranded RNA over 2000 base pairs in size. Upon activation, both RIG-I and MDA5 recruit IPS-1 (alternatively called MAVS), which ultimately activates NF-κB and IRF3 and the production of type I interferons. In addition, it was recently discovered that the NOD (nucleotide-binding oligomerization domain)-like receptor (NLR) NOD2 is activated by uncapped GU-rich single-strand RNA sequences [57]. Just like RIG-I and MDA5, NOD2 recruits IPS-1 to activate IRF3, which leads to the production of IFN-β.

Type I interferons are important in every aspect of the response against mRNA vaccines because they modulate processes like antigen expression, the function of APCs, and the differentiation of T cells [51]. It has further been shown that the production of type I interferons can be both beneficial and detrimental for mRNA vaccines depending on the timing of the “signal” [58]. It is thought that TCR signaling needs to happen before IFN signaling, to elicit the desired T cell response (STAT4 will be activated in the T cell), including CD8 T cell differentiation and proliferation to cytotoxic T cells [59,60,61]. When IFN signaling happens before the TCR is activated, STAT1 is activated and anti-proliferation and pro-apoptosis events are initiated [62,63]. Type I interferons are recognized by T cells through the IFNα/β-receptor (IFNAR) on the cell surface. Thus, while mRNA can be very potent, it can also shut down protein production through a host-cell defense mechanism to keep viruses from producing viral proteins.

It is therefore suggested that the interferon Type I response should be controlled, in order to improve translation of the mRNA vaccine candidate and consequently increase vaccine potency [32,44]. The innate immune response against exogenous mRNA can be minimized by avoiding the activation of TLR receptors, by using highly purified transcripts (complete removal of production byproducts, such as dsRNA and DNA template) and modified nucleosides (replacing uridine for pseudouridine) [25,62]. Another strategy is to suppress the receptors by co-expressing mRNA encoding immune evasion proteins, such as vaccinia virus proteins E3, K3, and B18 [64,65,66]. These proteins can locally and temporarily suppress PKR and IFN pathway activation and enhance expression of mRNA-encoded genes of interest. This option is especially interesting for self-amplifying mRNA because in vivo replicated mRNA cannot be purified or made with modified nucleosides.

#### 2.3.2. Cellular and Humoral Immune Responses to mRNA Vaccines

**Figure 4:**
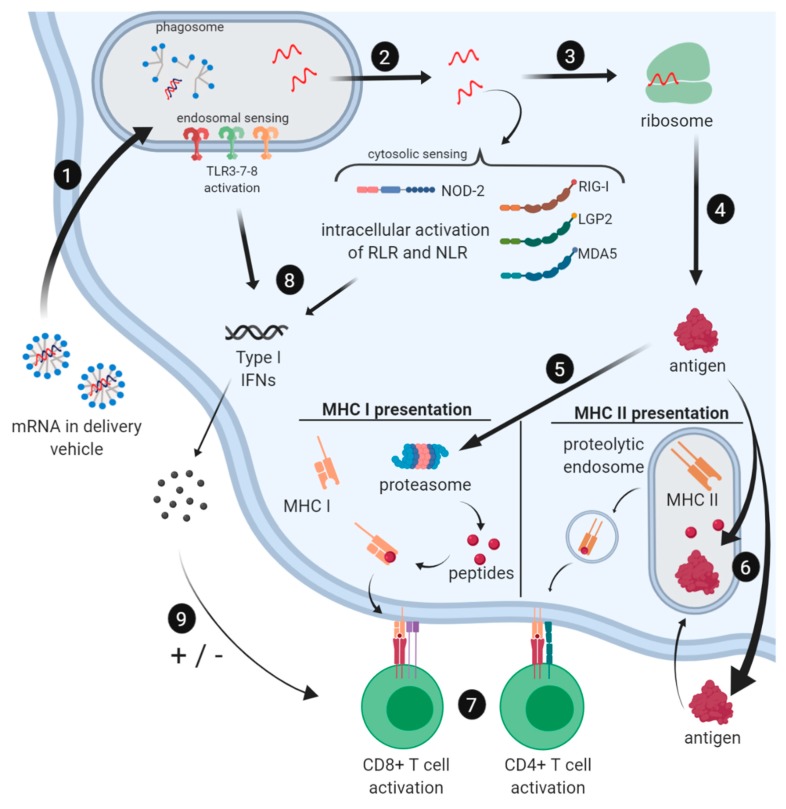
Messenger RNA-encoded vaccine antigen processing in antigen presenting cells (APCs). (1) mRNA encapsulated in the delivery vehicle is taken up by the host cell. After the delivery vehicle is digested, mRNA is recognized by Toll-like receptors (TLRs) and/or escapes from the phagosome (2). Different cytosolic pathogen recognition receptors can then recognize the mRNA. (3) mRNA is translated by the host’s ribosome and antigen is formed. (4) After the antigen is formed, it can be processed through different pathways. (5) The antigen is broken down to peptides by the host proteasome; peptides are accepted by major histocompatibility complex class I (MHC I). The MHC class I-peptide complex then travels to the cell membrane where it is presented to the immune system. (6) The antigen is secreted and ingested by an endosome or alternatively enters the endosome without secretion, achieved by adding signaling molecules and sequences. The antigen is then degraded by endosomal proteases and peptides are bound by major histocompatibility class II (MHC II). The MHC class II-peptide complex then travels to the cell membrane where it is presented to the immune system. (7) CD8+ and CD4+ T cell activation can be achieved through the presentation of the peptide on MHC class I and MHC class II, respectively. Co-stimulatory molecules and cytokines need to be present for successful activation. (8) TLR3-7-8 and NOD2, RIG-I, LGP2, and MDA5 can be activated by mRNA, subsequently triggering the production of type I interferons. (9) Secreted type I interferons can have a positive or negative effect on T cell activation. The activation level of the type I innate immune response triggered by mRNA can be controlled by the application of modified nucleosides, improved RNA purification, and low-immunogenic delivery systems.

Potent T cell responses through mRNA vaccination are achieved by targeting professional APCs, i.e., dendritic cells (DCs). Depending on the route of mRNA processing by the APCs, peptides derived from the mRNA can be presented on the major histocompatibility complex (MHC) class I or II of the APCs (Figure 4). In order to establish successful T cell activation and differentiation from naive T cells to effector cells, T cells must receive three different signals. Signal 1 involves the activation of the T cell receptor (TCR) by recognition of a peptide that is presented on the MHC of APCs. Signal 2 involves the binding of co-stimulatory molecules, such as CD80 and CD86, by CD28 on the T cells. Signal 3 consists of secreted cytokines that are then sensed by the T cell. A combination of all these signals will result in T cell activation and differentiation.

The humoral response is orchestrated by circulating antibodies secreted by B cells. It has been demonstrated that antigen-specific antibodies can be induced by mRNA vaccines [67,68,69,70,71]. B cells can be activated by circulating antigens binding to the B cell receptor (BCR). For mRNA vaccines, the availability of extracellular protein for B cell recognition can be enhanced by adding a secretion signal peptide to the RNA sequence or the addition of an MHC class II targeting sequence of a lysosomal or endosomal protein, such as LAMP (lysosomal-associated membrane protein) [72,73], which will allow the transfected cells to secrete the protein. Circulating proteins are taken up by B cells and peptides are displayed on the B cell’s MHC class II. T follicular helper (Tfh) cells, CD4+ T cells that have previously been activated by DCs displaying the MHC-II/peptide combination, will bind to the peptide-MHC class II of the B cells presenting the same peptide and subsequently release activation signals, including co-stimulatory molecules and cytokines (Figure 4). Antigen-experienced Tfh cells trigger the formation and maintenance of germinal centers (GCs) within secondary lymphoid organs, where B cell proliferation, class switching, and differentiation into memory B cells and antibody-secreting plasma cells take place [70]. It was shown in the literature that vaccination of mice with mRNA generates robust antigen-specific Tfh cell responses and an increased number of GC B cells, which results in long-lived high-affinity antibodies [71]. It was also proven that mRNA vaccine candidates can induce potent antibody responses against immunosubdominant targets [74], which are often important conversed regions on parasites and are ideal for vaccine development.

### 2.4. Advantages and Limitations of the mRNA Platform for Vaccine Development Against Parasitic Infections

While several vaccines against parasitic infections in humans have been developed at the pre-clinical stage, and some are even in early clinical trials, so far, only the malaria vaccine has made it to licensure. An effective vaccine should boost the immune response in a fashion that exceeds the “natural” innate and adaptive immune response. Most licensed vaccines also induce a memory immune response that provides long-term protection against infection. We will discuss the advantages and disadvantages of the mRNA vaccine development platform in this context.

#### 2.4.1. Production and Development

The production and purification process of IVT mRNA can be standardized, therefore avoiding the need for costly product-specific production and purification steps. While it faces the same regulatory requirements and needs for quality control as a recombinant protein [25], the mRNA purification process is less complicated [26]. Due to the standardization of the production and purification process, the development time for new vaccine candidates can be dramatically reduced, which allows for the rapid testing of more vaccine candidates by high-throughput screening [28,31]. The relatively simple, low-cost production process is a crucial benefit because regardless of the scientific and medical prospects, parasitic vaccines need to become accessible for people living in low-income countries [75].

On a lab-scale, new widely available kits allow for high-yield transcription reactions for the synthesis of capped RNA. The current costs of producing capped RNA are, however, still high at the larger scale, but, for example, more effective capping enzymes could lower cost [76]. Several companies and research institutes have built facilities for the GMP-grade large-scale synthesis (up to kilograms amounts) of capped, polyadenylated RNA. FDA-compliant enzymes and reagents to synthesize capped RNA have become available [29,77].

#### 2.4.2. Multivalent mRNA Vaccines

The mRNA platform allows for the simultaneous expression of multiple proteins, eliciting immunity to different epitopes from different targets [78,79,80]. Several antigens can either be combined into one mRNA sequence or a mixture of shorter RNA sequences, each translating into a different protein antigen that can be transfected together. The development of multivalent vaccines consisting of several antigens can even include a pan-parasitic approach, creating a vaccine, for example, targeting the multiple helminths that typically infect children in endemic areas. Additionally, not all individuals respond to the same parasitic antigens. Multivalent vaccines have a greater number of protective epitopes and thus should be efficacious in a greater proportion of the population. However, in multivalent vaccines, the optimal association or combination of antigens must be assessed to obtain synergistic effects. Additionally, mRNA vaccine mixtures may even encode immune evasive proteins [65,81] (see Section 2.3.1.) or co-stimulatory proteins that may further enhance the activation and differentiation of T cells [82].

#### 2.4.3. Strong Cellular Immune Responses

The immune profile triggered by mRNA vaccines has been discussed in detail in Section 2.3. When taken up by APCs, the mRNA vaccine induces a very strong T-cell response. However, with the use of signal markers, the immune response can be steered towards an increased humoral response. Because of the relatively simple option of multivalency, the platform also allows the combination of two mRNA antigens to be processed through different pathways; i.e., the mRNA sequences taken up by APCs without signal peptide will induce more T-cell responses, while other sequences that include a signal peptide will induce an antibody-mediated response.

#### 2.4.4. Stability

Previously, RNA was associated with low stability because of the omnipresence of RNases. However, the stability of IVT mRNA at −80 °C can be guaranteed for many months to years when the mRNA is properly capped and purified under sterile conditions [29,83,84]. In fact, a lyophilized mRNA vaccine for rabies has proven to be extremely thermostable [83]; its potency did not drop significantly over several months when the vaccine was stored at oscillating temperatures between 4 and 56 °C. This will be relevant as there is often a lack of a functional cold chain in many of the regions endemic for tropical parasites.

Once administered in vivo or in vitro, the stability of mRNA is limited. In mice, measurable levels of protein translation were found up to 10 days (5 µg dose), depending on the route of the delivery [71,85,86]. When using higher doses or with the application of self-amplifying RNA, RNA can be translated at high levels for several weeks [43,87]. The stability in vivo can be improved when the mRNA is encapsulated or linked to a protective delivery system [28,88,89,90]. However, it should be noted that from the vaccine safety perspective, the low half-life of the mRNA is typically regarded as an advantage.

#### 2.4.5. Safety Profile

The flipside of having a very potent product, such as mRNA, is the possibility of uncontrolled inflammatory reactions and possible toxicity. However, because of the transient character of mRNA and the relatively low doses (<100 µg) typically administered for vaccination, the risk is minimal. In addition, the inflammatory nature of mRNA can be further controlled by working with highly purified transcripts as well as modified nucleosides [32,34,67]. A report on the first in-human phase 1 clinical trial (NCT02241135) for an mRNA rabies vaccine (CV7201, CureVac, Tübingen Germany) concluded that the administration of the mRNA vaccine encoding a rabies virus glycoprotein was generally safe and reasonably tolerable [52]. Some limited local and systemic adverse reactions were noted; however, this is generally the case with most potent vaccine products. It will be interesting to see if the inflammatory nature of mRNA can be further controlled using an improved construct in an upcoming clinical study (NCT03713086). Unfortunately, a prostate cancer immunotherapy mRNA vaccine candidate (CV9104, CureVac, Tübingen, Germany), based on the same RNActive^®^ technology, has failed to meet the primary endpoint of improving overall survival in a phase IIb clinical trial (NCT01817738).

When compared to traditional vaccines, it is evident that using a gene construct coding for the antigen avoids the risks associated with whole-cell pathogens. In comparison to DNA vaccines, it is often noted that RNA cannot integrate into the host DNA (unless by a retrovirus), and from a regulatory perspective, mRNA vaccines are not considered a gene therapy product [91]. Furthermore, the manufacturing of IVT mRNA does not involve any animal-derived products. A side-by-side list of all the advantages and the disadvantages of traditional and novel oligonucleotide-based vaccine platforms is shown in Table 2.

## 3. Messenger RNA Vaccines Against Parasitic Infections

Despite different pre-clinical and even clinical studies existing that have shown the utility of mRNA vaccines against cancer, viral, and bacterial diseases, only a few studies have addressed parasitic diseases. Here, we discuss the few examples of mRNA vaccines against different parasitic infections.

### 3.1. Toxoplasma Gondii Infection

Chahal et al. have shown the possibility to achieve protective immunity against lethal doses of different infectious diseases using a dendrimer-RNA nanoparticle vaccine platform [79]. Modified dendrimer nanoparticles (MDNPs) were developed containing mRNA replicons encoding for antigens from either H1N1 influenza, Ebola virus, or the protozoan *Toxoplasma gondii*. In the case of *T. gondii*, self-amplifying mRNA constructs based on the Venezuelan equine encephalitis virus (VEEV) replicase proteins encoded for six different conserved *T. gondii* antigens, which are expressed by the protozoan throughout its lifecycle. Thirty-two days after a single vaccination, the mice were challenged with a lethal dose of *T. gondii* type II strain Prugniaud, and all vaccinated mice survived the lethal challenge, while the mice in the control group all died within 12 days.

Luo et al. demonstrated the potential of a self-amplifying mRNA vaccine candidate against *T. gondii* infection [92]. A lipid nanoparticle (LNP) was developed containing a self-replicating RNA vector RREP based on non-structural proteins of Semliki Forest virus (SFV) and an RNA sequence encoding for *T. gondii* nucleoside triphosphate hydrolase-II (NTPase-II). While vaccinating mice with the naked self-amplifying mRNA construct RREP-NTPase-II already induced strong specific immunoglobulin (IgG) titers and IFN-γ production, the immune responses were even more pronounced when the mRNA construct was delivered by the LNP. Mice that received the RREP-NTPase-II LNP vaccine candidate showed an increased survival time and survival rate versus control groups after being challenged with 10^3^ techyzoites of the RH strain. In addition, in a chronic animal model, in which mice were challenged with 20 tissue cysts of the PRU strain, mice who received RREP-NTPase-II and RREP-NTPase-II LNP showed a 46.4% and 62.1% reduction, respectively, in brain cysts when compared to the phosphate buffered saline (PBS) control group. The results indicated that vaccination with RREP-NTPase-II mRNA vaccine candidates can enhance resistance again acute and chronic challenges of *T. gondii*.

### 3.2. Malaria

Garcia et al. proved that protective immunity against malaria infection can be achieved by neutralizing the *Plasmodium* macrophage migration inhibitory factor (PMIF) utilizing the self-amplifying mRNA vaccine [93]. PMIF is an orthologue of the mammalian macrophage migration inhibitory factor (MIF) and secretion of PMIF by *Plasmodium* attenuates the host’s immune response. To improve host immunity, mice were vaccinated twice with a self-amplifying mRNA replicon encoding PMIF. It was observed that after vaccinations, PMIF specific CD4+ cells were increased, the anti-PMIF IgG titer increased 4-fold, and these antibodies blocked the pro-inflammatory action of PMIF without altering the functionality of host MIF. More importantly, a challenge with *Plasmodium* showed that vaccination with the PMIF mRNA enhanced the control of parasites and prevented re-infection.

### 3.3. Leishmania Donovani Infection

Recently, it was shown that protection against *Leishmania donovani* infection was accomplished by vaccinating mice with a heterologous mRNA—a subunit vaccine strategy. Duthie et al. developed a naked mRNA replicon encoding for the *LEISH-F2* gene [40]. When this F2-RNA was given as a prime vaccination and mice were boosted with the recombinant LEISH-F2 protein in glucopyranosyl lipid A in a stable oil-in-water emulsion (SLA-SE), a significant reduction in the parasite burden in the liver was observed. Other vaccination strategies, including homologous vaccination with either F2-RNA or LEISH-F2 SLA-SE, did not reduce parasite burdens compared to the control. In addition, the successful heterologous vaccine strategy was shown to induce very strong IFN-y secretions and antigen-specific Th1 responses by splenocytes, while vaccination with F2-RNA alone showed low antigen-specific Th1 responses and very low IgG responses and vaccination with LEISH-F2 SLA-SE alone showed slightly larger Th1 responses and strong IgG responses. These differences demonstrate the importance of how the antigen is produced and presented to the immune system.

## 4. Concluding Remarks and Prospects of New mRNA Vaccines for Parasitic Diseases

The IVT mRNA platform is currently one of the fastest growing vaccine technologies [26,94,95]. Similar to other oligonucleotide-based vaccine technologies, mRNA can be made using a standardized production process, allowing for multiple vaccine candidates to be screened within a reasonable time frame. The present-day costs of production are still high, however, the prospect of the low cost of standardized production may soon be realized through improved transcription and mRNA capping techniques and increased competition between GMP RNA production facilities. Different mRNA vaccine candidates can potentially be combined in multivalent vaccines. mRNA triggers a type-I innate immune response, leading to a strong CD8+ T cell response even without additional adjuvants. The immune reaction can also be steered towards antibody production by incorporating effective signal peptides. These features, mostly lacking in traditional vaccine platforms, make this platform very attractive for the development of vaccines against parasitic diseases. From a regulatory standpoint, mRNA products are less complex and safer than whole cell, DNA, or recombinant protein vaccines. Several mRNA vaccine candidates (for infectious diseases and cancers) have now found a pathway into the clinic and it will be interesting to study the immune reaction in humans. Hopefully, the additional potency of RNA versus DNA will push mRNA vaccines to surpass the clinical track record of DNA vaccines.

We foresee hundreds of new mRNA vaccine research results published in the next few years and expect several will be focused on parasitic diseases. We are currently developing an mRNA vaccine as part of our vaccine program against Chagas disease [96,97,98], a neglected tropical disease caused by the protozoan *Trypanosoma cruzi* resulting in cardiomyopathy and death. Although *T. cruzi* has been studied for decades now, no vaccine has been able to induce a 100% immune protection [87]. However, there is a consensus on what the properties of a protective vaccine would be. Ideally, an anti-*T. cruzi* vaccine should induce specific CD8+ T cells targeting conserved epitopes from both immunodominant and subdominant, or cryptic, *T. cruzi* antigens [99]. When applied as a therapeutic vaccine, a more balanced immune response is needed, in order to avoid triggering excessive host inflammation or autoimmunity [97]. Although there is a certain learning curve and the technology is in constant development, in our experience, the mRNA platform promises to be an effective vaccine tool to rapidly produce and screen different targets and induce potent CD8+ T cells responses against multiple antigens/epitopes of *T. cruzi* in a relatively easy manner. By these criteria, we conclude that the mRNA vaccine platform might become ideally suited for the development of neglected parasitic disease vaccines, on the condition that the costs of production continue to drop.

## Figures and Tables

**Table 1:** Prevalence, disability-adjusted life years (DALYs), and mortality of the most impactful parasitic diseases in 2017 [10].

Disease/Parasite	Prevalence	DALY’s	Deaths
Ascariasis *Ascaris lumbricoides*	447,008,998	860,833	3206
Trichuriasis *Trichuris trichiura*	289,617,741	212,664	N/A
Hookworm disease *Ancylostoma duodenale* and *Necator americanus*	229,217,130	845,010	N/A
Schistosomiasis *Schistosoma spp.*	142,788,542	1,431,447	8837
Malaria *Plasmodium spp.*	136,085,123	45,014,578	619,827
Chagas disease *Trypanosoma cruzi*	6,196,959	232,143	7853
Leishmaniasis *Leishmania sp.*	4,130,197	774,211	7527
Sleeping Sickness *Trypanosoma brucei*	4896	78,990	1364

**Table 2:** Advantages and disadvantages of different vaccine platforms for parasitic diseases.

Vaccine Platform	Advantages	Disadvantages
Killed/Attenuated Parasites	▪ Very potent ▪ Multivalent by nature ▪ Simple formulation, no adjuvants required	▪ Manufacturing challenge ▪ Requires stringent quality control ▪ Risk for infection
Subunit/Recombinant Protein	▪ Non-infectious ▪ Strong humoral response	▪ Need for additional immunostimulants (adjuvant) ▪ Need to develop new production process and stability assays for each new antigen ▪ Multivalent formulations can be challenging
Viral Vector	▪ Strong innate immune response ▪ Strong cellular and humoral responses	▪ Potential risk for infection ▪ Inflammation could cause risk for adverse reactions ▪ Pre-existing immunity against the vector ▪ Mixed results immunogenicity in humans
DNA	▪ Non-infectious ▪ Rapid development and production using standardized production pipeline ▪ Options for multivalency ▪ Strong T cell responses	▪ Poor immunogenicity in humans ▪ Potential risk at genetic integration
RNA	▪ Non-infectious ▪ Degradable and no risk for genetic integration ▪ Rapid development and production using standardized production pipeline ▪ Production free of any animal-derived products ▪ Options for multivalency ▪ Very potent innate immune response ▪ Strong T cell responses	▪ RNases can cause stability issues ▪ Inflammation could cause risk for adverse reactions ▪ Although becoming rapidly more affordable the current production costs are high
